# Cyclodipeptides Reversed Liver Damage and Adipose Tissue Dysfunction in a Chronic Obesity MASLD Rat Model by Remodeling White Adipocytes Toward a Beige-like Adipocyte Phenotype

**DOI:** 10.3390/molecules31142466

**Published:** 2026-07-15

**Authors:** Citlali Figueroa-Guzmán, Marlene Estefanía Campos-Morales, Lorena Martínez-Alcantar, Laura Hernández-Padilla, Elizabeth Sánchez-Duarte, Luis Alberto Sánchez-Briones, Jesús Salvador López-Bucio, Jesús Campos-García

**Affiliations:** 1 Instituto de Investigaciones Químico Biológicas, Universidad Michoacana de San Nicolás de Hidalgo, Morelia 58000, Michoacán, Mexico; 2 Programa Investigadoras e Investigadores por México, Secretaría de Ciencia, Humanidades, Tecnología e Innovación, Ciudad de Mexico 03940, Mexico; 3 Departamento de Ciencias Aplicadas al Trabajo, Universidad de Guanajuato, León 37150, Guanajuato, Mexico

**Keywords:** obesity, MASLD, steatosis, cyclodipeptides, adipocyte remodeling, beige-like adipose remodeling

## Abstract

**Background:** MASLD is a disorder linked to lipid metabolism and obesity, increasingly prevalent among sedentary people and leading to hepatic fibrosis. Cyclodipeptides (CDPs) have promising anti-obesogenic and liver-protective potential. **Methods:** CDP treatment was evaluated in a chronic MASLD model using female Wistar rats fed an obesogenic diet, with assessments of insulin resistance, glucose tolerance, liver damage, oxidative stress, and the expression of genes related to metabolic function. **Results:** MASLD CDP-treated rats showed low visceral adipose tissue (VAT) content, improved insulin responsiveness and glucose tolerance, reduced steatosis, and reversed oxidant stress and the *NRF2*, *GPX1*, and *GCLC* expression. Furthermore, MASLD-related dysregulation of genes involved in lipid metabolism was restored, including vLDL transport (*MTTP*, *APOB*, and *RASAL2*), β-oxidation (*PPAR-α*, *ACOX1*, and *FOXO1*), lipogenesis (*ACC1* and *SREBP 1C*), and fatty acid transport (*PSD3* and *CD36*). In accordance, genes of key signaling pathways were also restored, including *mTOR*, *TSC1*, and *TSC2*, along with fibrosis and inflammation *TGF-β*, *Fas*, *NF-κB*, and *IL-6*. In VAT of MASLD animals, crown-like structures and adiposity density were diminished by CDP treatment, with increased expression of genes associated with beige-like adipose tissue remodeling, including *PGC-1α*, *UCP1*, *NRF1*, *ATP6v1*, *CEBP-α*, *COX4i1*, *PPARγ*, and *CS*. Consistently, the UCP1 and PGC-1α protein expression was increased in the VAT of MASLD animals treated with CDPs. **Conclusions:** The anti-MASLD effects of CDPs were associated with reversal of key pathogenic markers in the liver and VAT, suggesting remodeling of white adipose tissue (WAT) toward a beige-like adipose tissue phenotype. The findings suggest that CDPs may modulate adipose tissue structure and adipogenesis, underscoring their therapeutic relevance for MASLD.

## 1. Introduction

Obesity is a major global public health challenge and a key risk factor for multiple metabolic disorders. This condition is characterized by a chronic imbalance in energy homeostasis, leading to systemic metabolic dysfunction that primarily involves adipose tissue (AT) and subsequently affects multiple organs, including the cardiovascular system, pancreas, muscle, and liver [1].

Metabolic-dysfunction-associated steatotic liver disease (MASLD) has emerged as the hepatic manifestation of metabolic syndrome and is strongly associated with obesity, insulin resistance, hypertension, dyslipidemia, and type 2 diabetes [2,3]. This condition can progress to cirrhosis and even hepatocellular carcinoma [4]. The development of MASLD is driven by alterations in lipid metabolism, including increased fatty acid flux to the liver, enhanced de novo lipogenesis (DNL), and impaired lipid export and oxidation [5].

Hepatocytes store excess lipids, such as triacylglycerides, in intrahepatic lipid droplets, a condition called steatosis [4]. In MASLD, lipotoxicity triggers liver damage through mechanisms such as endoplasmic reticulum stress and mitochondrial dysfunction, including alterations in the electron transport chain, leading to excessive ROS production. ROS and lipid peroxidation products impair hepatocyte function and viability while promoting stellate cell activation and differentiation, ultimately driving collagen production and extracellular matrix deposition characteristic of fibrosis [6].

Adipose tissue dysfunction is a key pathogenic event driving both the onset and progression of MASLD. When the capacity of white adipose tissue (WAT) to store excess energy is impaired, circulating free fatty acid levels rise due to dysregulated lipolysis and reduced lipid uptake. This altered lipid flux promotes ectopic fat deposition in peripheral organs, particularly the liver [7].

Adipose tissue is a highly dynamic and metabolically active organ composed primarily of WAT and brown adipose tissue (BAT), adipose depots that differ in structure and function [8]. WAT serves as the main site for energy storage, characterized by adipocytes with a single large lipid droplet and low mitochondrial content. In contrast, BAT specializes in dissipating energy through thermogenesis, driven by the UCP1 protein, which uncouples oxidative phosphorylation from ATP production. Beyond these classical depots, adipose tissue exhibits remarkable plasticity, allowing the emergence of beige adipocytes within WAT in response to specific stimuli. These cells share morphological and functional features with brown adipocytes, including multilocular lipid droplets, high mitochondrial content, and UCP1 expression, thereby contributing to increased energy expenditure. This process, known as adipose tissue browning, has been proposed as a key adaptive mechanism to counteract metabolic imbalance [9].

Under chronic energy surplus, as in obesity, adipose tissue undergoes significant structural and functional changes. White adipocytes become hypertrophic, leading to local hypoxia, cellular stress, and the recruitment of immune cells, particularly macrophages, which promote a pro-inflammatory, insulin-resistant environment [10].

Given the high global prevalence of MASLD and the lack of effective pharmacologic treatments, there is an urgent need to identify strategies that target the underlying metabolic dysfunction [11]. In this context, restoring adipose tissue function is a promising approach to re-establish metabolic homeostasis and limit hepatic lipid accumulation.

Microbe-derived metabolites have emerged as modulators of metabolic pathways. Cyclodipeptides (CDPs), the simplest and most prevalent cyclic peptides, play roles in cell-to-cell communication and in switching between virulence and symbiosis, and exhibit antibacterial, antifungal, antiviral, and antitumor properties [12,13,14]. The CDP mixture isolated from the *P. aeruginosa* PAO1 culture medium, composed mainly of cyclo(L-Pro-L-Val), cyclo(L-Pro-L-Leu), cyclo(L-Pro-L-Phe), and cyclo(L-Pro-L-Tyr), shows cytotoxic effects on the HeLa cancer cell line by blocking the PI3K/AKT/mTOR signaling pathway [15]. Transcriptomic analysis of HeLa cells treated with bacterial CDPs revealed significant changes in gene expression profiles. Notably, approximately 30% of the differentially expressed genes were associated with energy metabolism [16]. Furthermore, CDPs modulate the expression of genes recently linked to the development of MASLD, including *PSD3* [17] and *RASAL2* [18]. Recently, treatment with cyclo(L-His-L-Pro) in a fibrotic MASLD mouse model has been shown to decrease hepatic fat accumulation and reduce pro-inflammatory cytokines via ERK signaling [19]. A previous study in Wistar rats fed a high-fat diet (HFD/CAF) showed that treatment with bacterial CDPs reduced hepatic lipid content, decreased inflammatory marker expression, and improved aquaporin levels (AQP1, AQP5, AQP8, and AQP9) (Figueroa-Guzmán et al., in review). However, the mechanisms underlying these effects remain poorly understood. Therefore, this study aimed to evaluate the impact of CDPs in a chronic MASLD model and to explore the mechanisms underlying their metabolic effects.

## 2. Results

### 2.1. CDPs Isolation, Chronic Obesogenic Model in Rats, and Anti-Obesogenic CDP-Treatment

**Figure 1:**
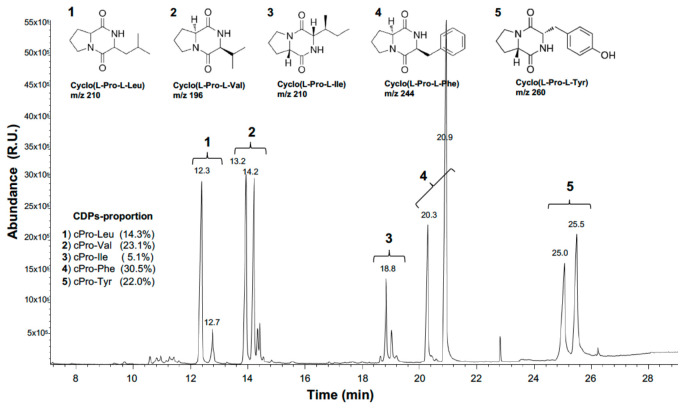
Chromatographic analysis of the CDPs isolated from cultures of the *P. aeruginosa* PAO1 strain. The GC-MS analysis of the CDP mixture showed that it was composed of isomers, with the following proportions: **1.** cyclo(L-Pro-L-Leu) (14.3%), *m*/*z* = 210: 70(100), 154(98), 41(32), 43(26), 86(25), 55(18), 69(18), 125(10), 124(9), 139(6), 167(4), 195(2), 210(1). **2.** cyclo(L-Pro-L-Val) (23.1%), *m*/*z* = 196: 154(100), 70(65), 125(30), 72(26), 41(15), 55(10), 138(6), 110(5), 196(3). **3.** cyclo(L-Pro-L-Ile) (5.1%), *m*/*z* = 210: 154(100), 70(65), 57(25), 86(20), 125(15), 99(7), 113(5), 138(4), 167(2), 181(2), 197(1), 210(1). **4.** cyclo(L-Pro-L-Phe) (30.5%), *m*/*z* = 244: 125(100), 244(55), 153(48), 91(45), 70(44), 120(17), 103(10), 41(5), 55(2), 201(2), 227(1), and **5.** cyclo(L-Pro-L-Tyr) (22.0%), *m*/*z* = 260: 154(100), 107(50), 70(25), 260(10), 125(3), 207(2); representing >95% purity in the mixture. CDP structures obtained from the NIST-2023 library and mass fragmentation profiles are shown.

In a previous study, we reported that the mixture of CDPs isolated from *P. aeruginosa* PAO1 cultures was composed mainly of cyclo(L-Pro-L-Val), cyclo(L-Pro-L-Leu), cyclo(L-Pro-L-Phe), and cyclo(L-Pro-L-Tyr) [15]; however, in the present study, the CDPs mixture was enriched by resin interaction (see Materials and Methods). The GC-MS analysis of the CDPs mixture showed that it was composed of cyclo(L-Pro-L-Leu) (14.3%), cyclo(L-Pro-L-Val) (23.1%), cyclo(L-Pro-L-Phe) (30.5%), and cyclo(L-Pro-L-Tyr) (22.0%), and additionally, cyclo(L-Pro-L-Ile) was identified at 5.1% (Figure 1). The CDPs proportions represent >95% of the compounds contained in the extraction.

**Figure 2:**
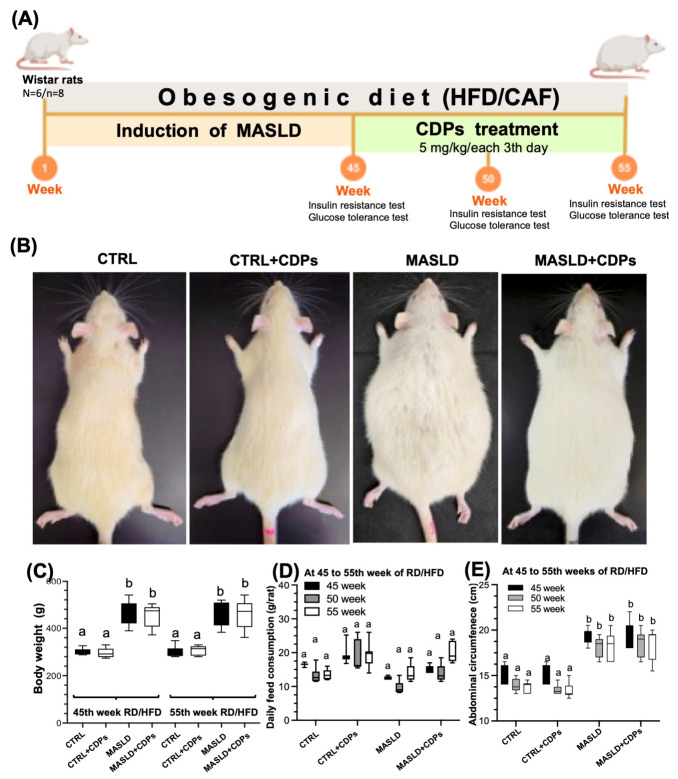
Evaluation of murinometric parameters in rats with MASLD treated with CDPs. (**A**) Twenty-eight female Wistar rats (6 weeks old) were enrolled in an experimental anti-MASLD therapeutic study. Nomenclature of animals: CTRL, healthy control animals (*n* = 6); CTRL + CDPs, healthy control animals administered CDPs at 5 mg/kg every three days for 10 weeks (*n* = 6); MASLD, animals that developed MASLD by feeding with an obesogenic HFD/CAF diet (*n* = 8); MASLD + CDPs, animals with MASLD administered CDPs at 5 mg/kg every three days for 10 weeks (*n* = 8). (**B**) Representative photographs of animals from each group at the end of CDPs treatment. (**C**) Body weight of animals at the 45th week and at the end of the therapeutic study (55th week). (**D**) Mean daily consumption per rat for each group on regular diet (RD) or hypercaloric diet (HFD/CAF) during the therapeutic procedure (from 45th week to 55th week). (**E**) Abdominal circumference measured from the 45th to the 55th weeks of the therapeutic procedure. Bars represent means ± SD, *n* = 6–8 per group. (**C**–**E**) Statistical analysis was performed using a mixed-effects model (REML) for repeated measures, followed by Tukey’s multiple-comparisons. Data are presented as mean ± SD. Different lowercase letters indicate significant differences among groups (*p* < 0.05).

On the other hand, a chronic MASLD model was established using an HFD/CAF diet administered for 55 weeks to promote obesity-associated liver fibrosis (Figure 2A). At 45 weeks of HFD/CAF feeding, animals developed obesity, characterized by increased body weight and abdominal circumference compared with RD controls (Figure 2B–E). At this stage, CDPs administration was initiated and continued for 10 weeks (weeks 45 to 55).

Following treatment, the CTRL and CTRL + CDPs groups did not differ significantly in body weight or feed intake. However, animals fed the obesogenic diet from the 45th to the 55th week maintained stable body weight, with altered daily feed intake in the MASLD group, as in the MASLD + CDPs group (Figure 2C,D). At the end of treatment (55th week), the MASLD groups showed a significant increase in weight and abdominal circumference compared with the RD-fed animals, suggesting the development of features consistent with chronic MASLD. In addition, CDP treatment did not significantly reduce body weight or abdominal circumference (Figure 2E).

**Figure 3:**
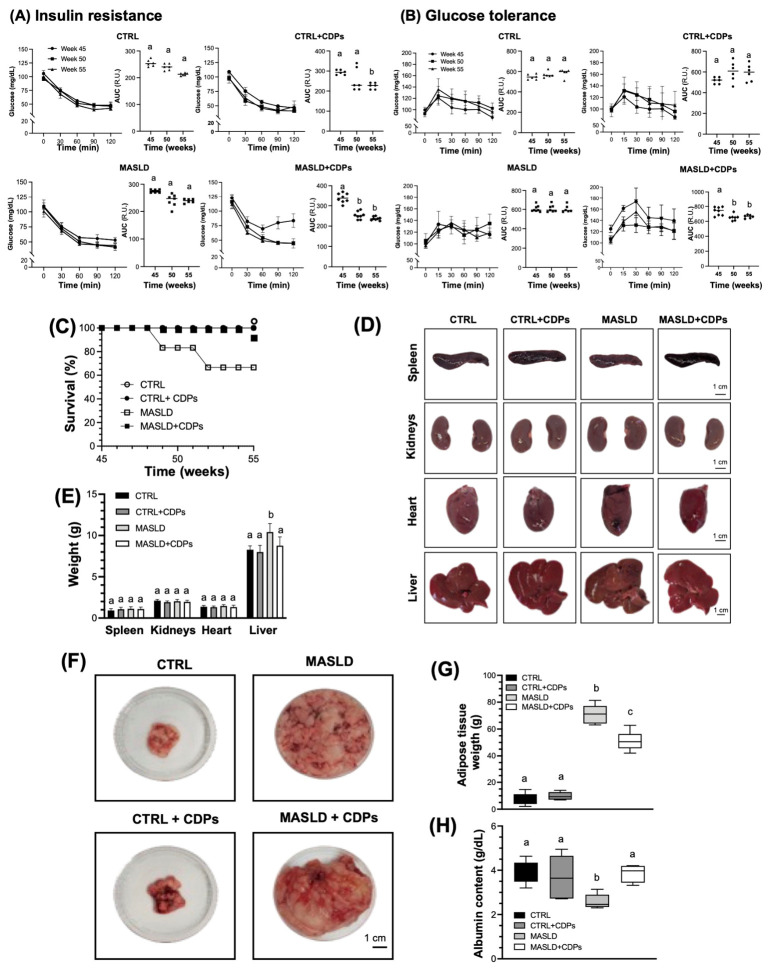
Longitudinal evaluation of insulin sensitivity and glucose tolerance tests, survival, and macroscopic analysis of organs in rats with MASLD developed and treated with CDPs. (**A**,**B**) Insulin sensitivity and glucose tolerance tests were performed during the 45th, 50th, and 55th weeks of CDPs treatment. The areas under the curve (AUC) for each animal group are shown on the right; AUC values were analyzed using a mixed-effects model (REML). Sample sizes at week 45 were CTRL (*n* = 6), CTRL + CDPs (*n* = 6), MASLD (*n* = 8), and MASLD + CDPs (*n* = 8). (**C**) Survival percentage of the animal groups throughout the experimental therapeutic study; two animals from the MASLD group died at weeks 48 and 52, resulting in *n* = 6 at the final procedure (55th week). Missing individuals who died during the study were accounted for in the statistical analysis using a mixed-effects model. (**D**) Representative images of organs, including the spleen, kidneys, heart, and liver, for each experimental group. Scale bar = 1 cm. (**E**) Organ weights. (**F**) Representative photographs of visceral adipose tissue from one animal of each group, scale bar = 1 cm. (**G**) Average visceral adipose tissue weight for each animal group. (**H**) Serum albumin levels. (**E**,**G**,**H**) Statistical analysis was performed using one-way ANOVA, followed by Tukey’s post hoc test; bars represent the means ± SD, *n* = 6–8 per group; significant differences (*p* < 0.05) are indicated with different lowercase letters.

To evaluate systemic metabolic dysfunction during treatment progression, insulin resistance and glucose tolerance tests were performed at week 45 (treatment initiation), week 50 (mid-treatment), and week 55 (end of treatment). Animals receiving CDPs showed progressive improvement in insulin responsiveness throughout the treatment period. Compared with baseline, the MASLD + CDPs group exhibited lower insulin tolerance test AUC values at weeks 50 and 55, whereas untreated MASLD animals did not show comparable improvement (Figure 3A). Consistent with these observations, mixed-effects analysis revealed a significant interaction between time and treatment (*p* = 0.0002). Similarly, during the longitudinal therapeutic period (45th to 55th weeks) of glucose tolerance monitoring, differences in AUC values were observed between the MASLD and MASLD + CDPs experimental groups. Although the overall effect of time was not significant, a mixed-effects analysis revealed a significant interaction between time and treatment (*p* = 0.001). In particular, MASLD + CDPs animals showed lower glucose tolerance AUC values at week 55 than at baseline (Figure 3B). Interestingly, improved insulin sensitivity was also observed in healthy animals treated with CDPs.

During the chronic experimental period, all CTRL, CTRL + CDPs, and MASLD + CDPs animals survived to study completion, whereas the untreated MASLD group had a 25% mortality rate (2/8 rats) (Figure 3C). At the experimental endpoint, the remaining animals were euthanized for tissue collection. Post-mortem analyses revealed no significant differences in spleen, kidney, or heart weights among groups; however, hepatomegaly and macroscopic fibrotic lesions were observed in MASLD animals and were attenuated following CDPs treatment (Figure 3D,E). The MASLD group exhibited a significant accumulation of visceral adipose tissue (average of 70 g); in contrast, visceral adipose tissue weight was reduced in the MASLD + CDPs group (average of 50 g). The CTRL groups showed similar adipose tissue weight (average of 10 g) (Figure 3F,G). In addition, serum albumin levels, which were reduced in MASLD animals, were restored after CDPs administration (Figure 3H).

### 2.2. Steatosis and Fibrosis in the Livers of Rats with MASLD Are Alleviated by CDPs Treatment

**Figure 4:**
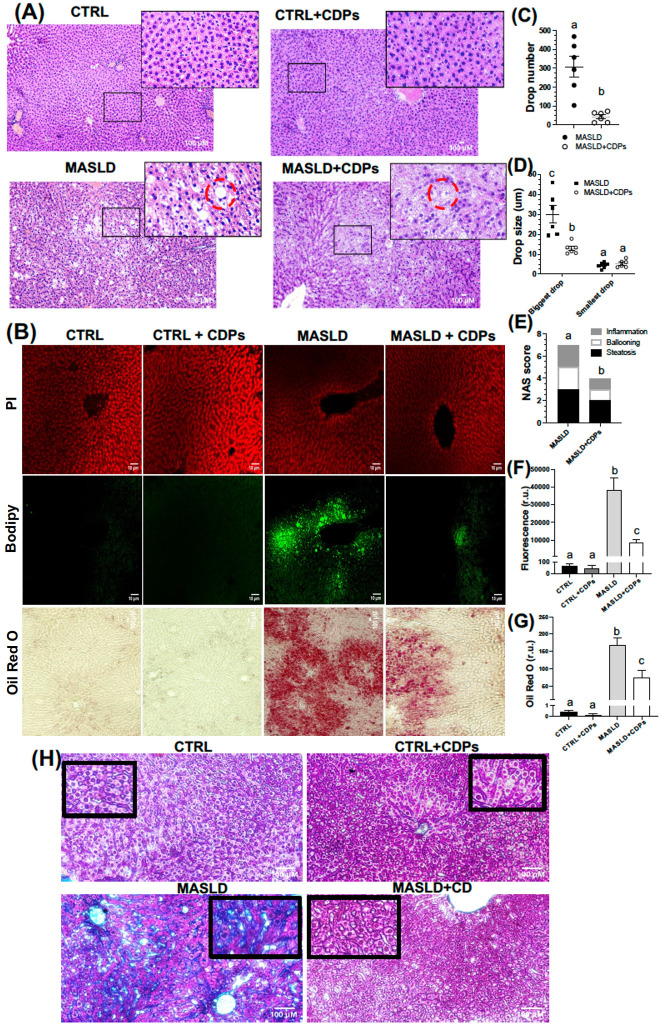
Histological analysis of liver tissue from rats with MASLD treated with CDPs. (**A**) Representative optical microscopy images of hematoxylin and eosin-stained liver histological sections (10× magnification, with enlargements). Red circles highlight macrovesicular steatosis in the MASLD group, whereas the MASLD + CDPs group exhibits microvesicular steatosis. Liver samples from six animals per group (*n* = 6) were processed for H&E staining. For quantitative analysis of parameters (**C**,**D**), fifteen microscopic fields per liver sample of each animal were evaluated. (**B**) Representative confocal images of liver tissue sections stained with propidium iodide (PI), Bodipy, and Oil Red O (*n* = 6). (**C**–**G**) Quantification of parameters shown in images (**A**,**B**). (**C**) Lipid droplet number. (**D**) Lipid droplet size (μm). (**E**) NAS. (**F**) Fluorescence intensity of Bodipy staining. (**G**) Color intensity of Oil Red O staining. (**H**) Representative Masson’s trichrome-stained liver sections (20× magnification, black squares show image magnification); collagen fibers indicative of fibrosis are shown in blue. Animal groups are defined in Figure 2. Bars represent the means ± SD, *n* = 6 animals per group; fifteen fields analyzed per animal. Statistical analysis was performed using one-way ANOVA, followed by Dunnett’s post hoc test. Different lowercase letters indicate significant differences (*p* < 0.01).

H&E staining of liver tissue revealed marked lipid droplet accumulation, macrovesicular steatosis, hepatocyte ballooning, inflammatory foci, and an increased NAS in the MASLD group compared with RD-fed animals (Figure 4A–E). Importantly, these histopathological alterations were significantly reduced in the MASLD + CDPs group, indicating improvement in liver damage following CDPs treatment.

Lipid accumulation was further confirmed by Bodipy and Oil red-O staining. The CTRL groups showed minimal lipid accumulation, whereas the MASLD group exhibited abundant lipid deposits. In contrast, the MASLD + CDPs group showed markedly lower lipid accumulation (Figure 4B,F,G).

Additionally, Masson’s trichrome staining showed increased collagen deposition along hepatic sinusoids in MASLD animals, whereas CDPs treatment reduced collagen fiber accumulation. Minimal collagen staining was observed in CTRL groups (Figure 4H).

### 2.3. Expression of Genetic Components Involved in Hepatic Fibrosis and Lipid Metabolic Pathways in the Livers of Rats with MASLD

**Figure 5:**
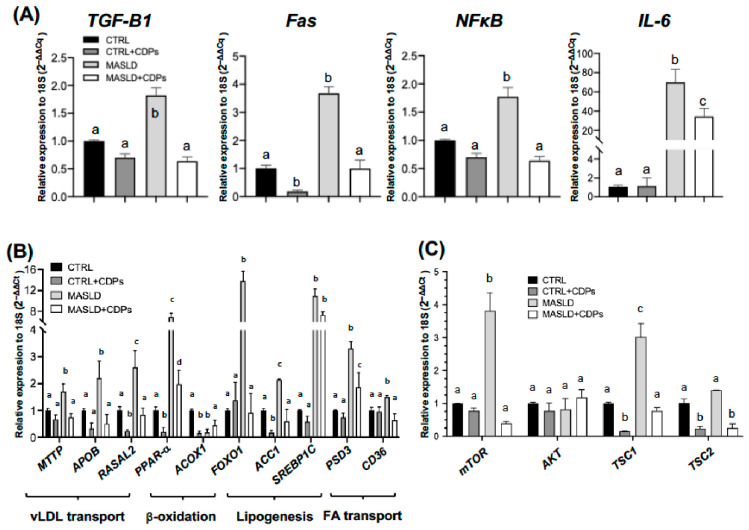
Gene expression of metabolic pathway components in the livers of rats with MASLD treated with CDPs. (**A**) Relative expression of genes determined by RT-qPCR in the livers of MASLD animals treated with CDPs, related to fibrosis (*TGF-β* and *Fas*) and inflammation (*NFκB* and *IL-6*). (**B**) Relative expression of genes in liver tissue related to lipid metabolism (*MTTP*, *APOB*, *RASAL2*, *PPAR-α*, *ACOX1*, *FOXO1*, *ACC1*, *SREB1C*, *PSD3*, and *CD36*). (**C**) Relative expression of genes in liver tissue of the mTOR pathway (*mTOR*, *AKT*, *TSC1*, and *TSC2*). Three liver samples from six animals per experimental group (*n* = 6) were pooled, and mRNA was extracted and RT-qPCR was performed (M&M section). Gene expression values were normalized using the housekeeping *18S* gene. Animal groups are defined in Figure 2. Bars represent the means ± SEM of three repetitions per group. Statistical analysis was performed using one-way ANOVA, followed by Tukey’s post hoc test. Different lowercase letters indicate significant differences (*p* < 0.05).

Hepatic expression of pro-fibrotic markers was quantified by RT-qPCR. The MASLD group exhibited increased hepatic expression of *TGF-β* and *Fas*, which was significantly reduced in the MASLD + CDPs group, reaching levels comparable to those of the CTRL groups (Figure 5A). Additionally, the MASLD group exhibited the highest expression of *NFκB* and *IL-6*, inflammatory markers, which were significantly reduced in the MASLD + CDPs group to levels similar to controls (Figure 5A).

We examined the expression of key genes involved in VLDL assembly and secretion, β-oxidation, DNL, and fatty acid transport (Figure 5B). In animals with MASLD, significant upregulation was observed in genes associated with VLDL synthesis and export, including *MTTP*, *APOB*, and *RASAL2*. This upregulation was reversed in the MASLD + CDPs group, restoring expression to levels comparable to those in the CTRL groups. Interestingly, in both the CTRL and MASLD groups, CDP treatment reduced expression of *APOB* and *RASAL2* compared with untreated groups (Figure 5B).

Regarding genes associated with the β-oxidation pathway, hepatic *PPARα* expression was significantly higher in the MASLD group than in the CTRL group, whereas CDPs administration significantly reduced PPARα expression in the MASLD + CDPs group (Figure 5B). In contrast, *ACOX1* expression was markedly reduced in the MASLD group and partially restored in the MASLD + CDPs group. To further assess expression of genes involved in DNL, *FOXO1*, *SREBP-1C*, and *ACC1* were examined (Figure 5B). The MASLD group exhibited elevated expression of all three genes compared with the CTRL group, while the CDPs administration in the MASLD group significantly reduced their expression. Importantly, *ACC1* expression was also reduced in the CTRL + CDPs group compared with the untreated control. Finally, expression of the *PSD3* and *CD36* genes, which are involved in fatty acid transport, was significantly upregulated in the MASLD group and was reversed in the MASLD + CDPs group to levels similar to those of the CTRL groups (Figure 5B).

On the other hand, analysis of gene expression associated with the PI3K/AKT/mTOR pathway in the livers of animals with MASLD showed that *mTOR* mRNA expression was significantly increased compared with the CTRL and CTRL + CDPs groups; importantly, this increase was significantly attenuated following CDPs treatment (Figure 5C). In addition, similar behavior was observed in *TSC1* gene expression; however, no significant differences were observed in *AKT* and *TSC2*. Unexpectedly, the *TSC2* gene was downregulated by CDPs treatment (Figure 5C).

### 2.4. Oxidative Stress in the Livers of Rats with MASLD Is Alleviated by CDPs Treatment

**Figure 6:**
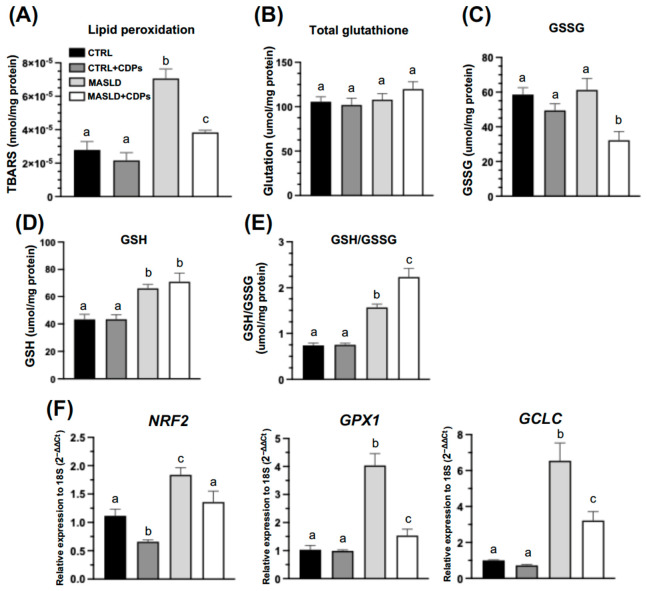
Determination of oxidative stress in the livers of rats with MASLD treated with CDPs. (**A**) Lipid peroxidation was measured in liver tissue as TBARS levels. (**B**) Assessment of total glutathione. (**C**) Oxidized glutathione levels (GSSG). (**D**) Reduced glutathione levels (GSH). (**E**) GSH/GSSG ratio. Liver samples from six animals per experimental group (*n* = 6) were pooled and homogenized for compound determination in samples by triplicate. (**F**) RT-qPCR was used to determine the relative expression of the *NRF2*, *GPX1*, and *GCLC* genes, normalized to the housekeeping *18S* gene. Liver samples from six animals (*n* = 6) per experimental group were pooled, and mRNA was extracted, and RT-qPCR was performed in triplicate (M&M section). Animal groups are defined in Figure 2. Bars represent the means ± SEM of three repetitions per group. Statistical analysis was performed using one-way ANOVA, followed by Tukey’s post hoc test. Different lowercase letters indicate significant differences (*p* < 0.05).

The TBARS assay was used to determine hepatic lipid peroxidation. As expected, animals in the MASLD group showed elevated TBARS levels compared to those in the CTRL and CTRL + CDPs groups (Figure 6A). CDPs administration significantly reduced TBARS levels in the MASLD + CDPs group.

To assess redox homeostasis, hepatic glutathione status was evaluated. Total glutathione levels did not differ significantly among groups (Figure 6B). In contrast, oxidized glutathione (GSSG) levels were lowest in the MASLD + CDPs group (Figure 6C). A significant increase in the reduced glutathione (GSH) fraction was observed in the MASLD and MASLD + CDPs groups (Figure 6D). The GSH/GSSG ratio was significantly higher in the MASLD group than in the CTRL animals and was highest in the MASLD + CDPs group (Figure 6E).

To further investigate oxidative stress induction, hepatic mRNA expression of *NRF2*, *GPX1*, and *GCLC* was measured. The results showed a significant increase in expression of the *NRF2*, *GPX1*, and *GCLC* genes in the MASLD group compared with the CTRL groups. In the MASLD + CDPs group, expression of these genes was significantly reduced (Figure 6F).

### 2.5. Dysfunction in the Visceral Adipose Tissue of Rats with MASLD Is Reversed by CDPs Treatment

**Figure 7:**
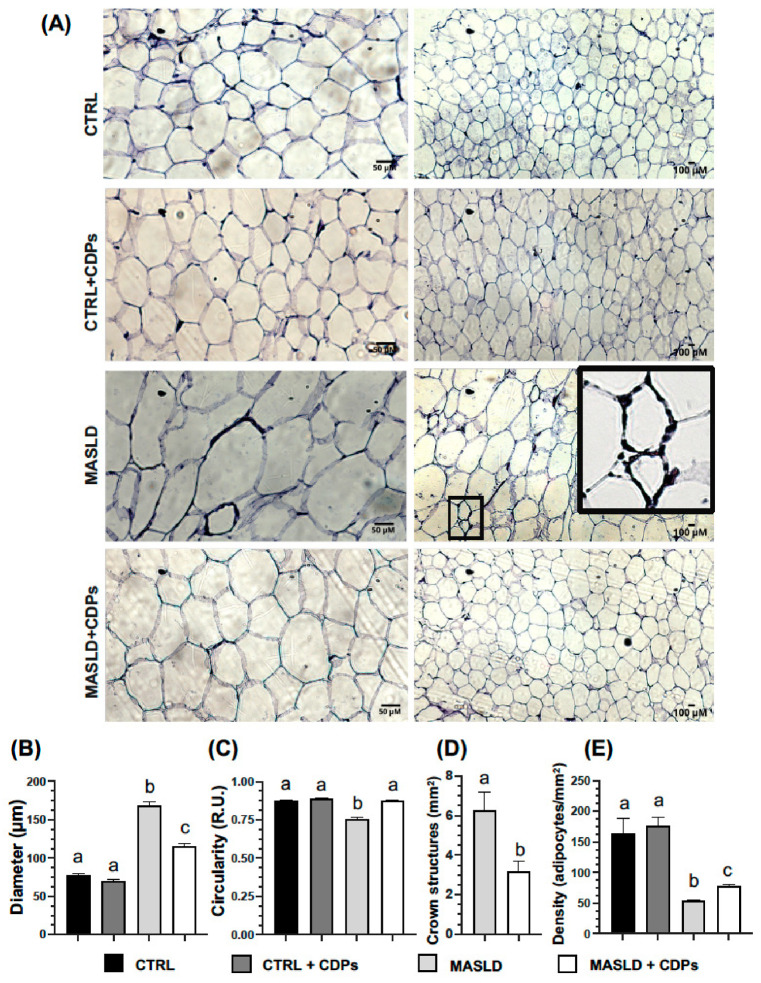
Histological analysis of visceral adipose tissue from rats with MASLD treated with CDPs. (**A**) Representative optical microscopy images of hematoxylin and eosin-stained histological sections of visceral adipose tissue (10× and 40× magnification). The square highlights crown-like structures in adipocytes, indicative of macrophage infiltration in the visceral adipose tissue of the MASLD group. (**B**–**E**) Three VAT samples from six animals per group (*n* = 6) were dissected and processed for H&E staining. For analysis, twelve representative microscopic fields from each of six animals (*n* = 6) in each experimental group were assessed to quantify adipocyte parameters. (**B**) Adipocyte diameter. (**C**) Adipocyte circularity. (**D**) Number of crown-like structures in the visceral adipose tissue per mm^2^. (**E**) Density of adipocytes per mm^2^ of tissue. Bars represent the means ± SE, *n* = 6 animals per group. Statistical analysis was performed using one-way ANOVA, followed by Tukey’s post hoc test. Different lowercase letters indicate significant differences (*p* < 0.05).

**Figure 8:**
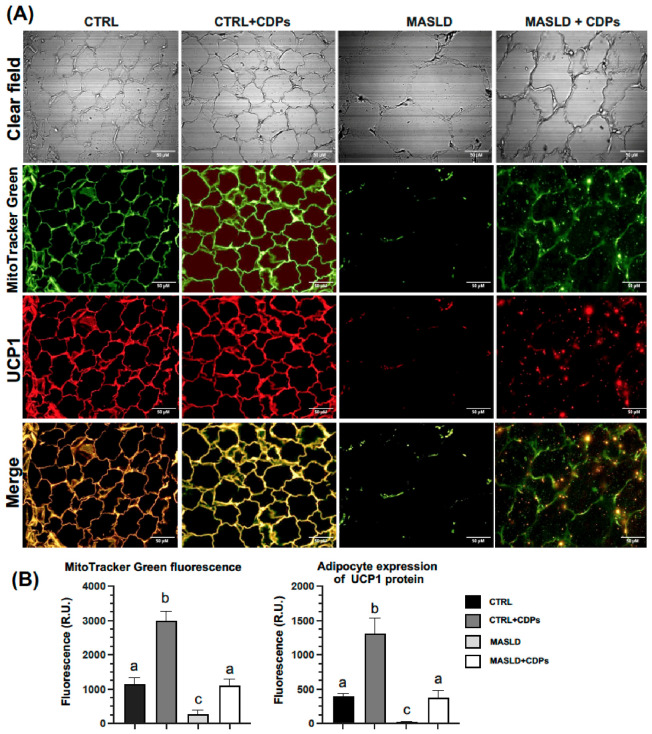
Confocal analysis of visceral adipose tissue from rats with MASLD treated with CDPs. (**A**) Representative confocal microscopy images of visceral adipose tissue sections stained with Propidium Iodide (PI), MitoTracker Green, and anti-UCP1 antibody, with clear-field images shown (20× magnification). Scale bar = 50 μm. Animal groups are defined in Figure 2. (**B**) Quantification of fluorescence intensity using MitoTracker Green staining and UCP1 immunodetection. VAT samples from six animals per animal group (*n* = 6) were dissected and processed for staining. Fifteen representative microscopic fields acquired from the six animals in each experimental group were analyzed to quantify fluorescence. Bars represent the means ± SEM, *n* = 6 animals per group. Statistical analysis was performed using one-way ANOVA, followed by Tukey’s post hoc test. Different lowercase letters indicate significant differences (*p* < 0.05).

In animals that developed MASLD, adipocytes in visceral adipose tissue (VAT) had an average diameter >160 µm, and their circularity was more irregular than in the CTRL groups (Figure 7). Importantly, the MASLD group treated with CDPs had adipocytes with an average diameter of 110 µm and circularity similar to that of the CTRL groups. As shown, the adipocytes of the CTRL and CRTL + CDPs groups had an average diameter of 70–75 µm, with no significant differences in cell circularity (Figure 7A–C). In addition, crown-like structures in adipocytes, indicative of macrophage infiltration, were observed at a significantly higher number per unit tissue area in the MASLD group than in the MASLD + CDPs group (Figure 7A,D); this correlated with adipocyte density (Figure 7E). As expected, in the CTRL groups, no crown-like structures were observed, and adipocyte density was unchanged (Figure 7A,E). VAT stained with MitoTracker Green showed mitochondrial-associated fluorescence signals that colocalized with UCP1 immunodetection (Figure 8A,B). The highest levels of UCP1 and MitoTracker were found in the CTRL + CDPs group; however, a significant decrease in fluorescence was observed at the adipocyte contours of the MASLD group, which was partially restored in the MASLD + CDPs tissue.

### 2.6. Expression of Mitochondrial Genes in the Visceral Adipose Tissue of Rats with MASLD Treated with CDPs

**Figure 9:**
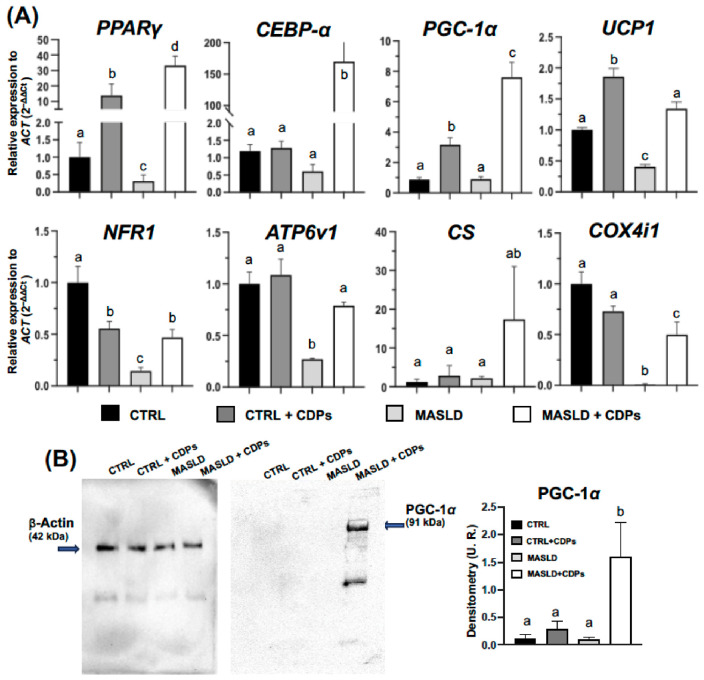
Gene and protein expression of components related to mitochondrial biogenesis in the visceral adipose tissue of rats with MASLD treated with CDPs. (**A**) Relative expression of genes determined by RT-qPCR related to mitochondrial biogenesis (*PPAR-γ*, *CEBP-α*, *PGC-1α*, and *UPC1*) and mitochondrial function and energy generation (*NFR1*, *ATP6v1*, *CS*, and *COX4i1*). All values were normalized using the housekeeping *ACT* gene. Animal groups are defined in Figure 2. VAT samples from six animals (*n* = 6) per experimental group were pooled, and mRNA was extracted and RT-qPCR was performed in triplicate (M&M section). Bars represent the means ± SEM, of three repetitions per group. Statistical analysis was performed using one-way ANOVA, followed by Tukey’s post hoc test; SEM values are shown as bars, and significant differences (*p* < 0.05) are indicated with different lowercase letters. (**B**) Western blot assay of protein extracts obtained from pooled VAT from six animals (*n* = 6) per experimental group, which was homogenized and subjected to immunodetection performed in three independent repetitions. Representative immunoblot showing total PGC-1 and β-actin expression, developed using anti-PGC-1 and anti-β-actin antibodies. At right, densitometric analyses of the immunodetection using Image J, normalized to β-actin expression. Bars represent the means ± SEM of three repetitions per group. Statistical analysis was performed using one-way ANOVA, followed by Tukey’s post hoc test. Different lowercase letters indicate significant differences (*p* < 0.05).

The essential roles of the PPARγ receptor and CEBPα in fibroblast-to-adipocyte differentiation were determined. Increased expression of *PPARγ* and *CEBPα* in VAT was observed in the MASLD + CDPs group compared with the MASLD group. Interestingly, only *PPARγ* was significantly increased in the CTRL + CDPs group, whereas *CEBPα* was not (Figure 9A).

The expression of *PGC-1α* gene increased in the VAT of animals treated with CDPs, with the highest levels in the MASLD + CDPs group (Figure 9A). Additionally, *UCP1* expression was significantly inhibited in the MASLD group but was restored by CDP treatment, reaching levels comparable to those in the CTRL group. Interestingly, both *PGC-1α* and *UCP1* expression were significantly increased in the CTRL + CDPs group.

Additionally, in the MASLD group, expression of the *NRF1*, *ATP6v*, *CS*, and *Cox4i1* genes was significantly inhibited but reversed by CDP treatment. In the CTRL groups, no significant changes in expression were observed (Figure 9A).

PGC-1α protein expression in VAT was further confirmed by immunodetection, which showed that it was diminished in the MASLD and control groups but significantly overexpressed in the MASLD + CDPs group (Figure 9B).

## 3. Discussion

This study demonstrates that administering an enrichment mixture of CDPs isolated from *P. aeruginosa*, comprising cyclo(L-Pro-L-Leu), cyclo(L-Pro-L-Val), cyclo(L-Pro-L-Phe), cyclo(L-Pro-L-Tyr), and cyclo(L-Pro-L-Ile) in a 14:23:30:22:5 ratio (Figure 1), exerts multiple beneficial therapeutic effects in a chronic MASLD model. Using a 55-week experimental design, the study reproduced the progression of human MASLD from hepatic steatosis to advanced fibrosis and showed that CDPs induced coordinated transcriptional changes in pathways associated with insulin sensitivity, hepatic lipid metabolism, and VAT remodeling. Importantly, CDPs induced morphological and transcriptional features consistent with a beige-like adipocyte phenotype, suggesting enhanced adipose tissue plasticity.

MASLD is a multifactorial disease in which VAT dysfunction plays a major role in the progression of liver injury [20]. Pathological adipocyte expansion, chronic inflammation, and loss of metabolic plasticity promote a sustained flux of fatty acids, pro-inflammatory adipokines, and lipotoxic signals to the liver, thereby contributing to steatosis, insulin resistance, and fibrosis [21]. In this context, our findings suggest that CDPs exert therapeutic effects on both the liver and VAT, supporting the restoration of adipose–liver crosstalk during the chronic progression of MASLD.

Body weight gain is strongly associated with obesity-related metabolic dysfunction and MASLD progression [22]. In our experimental MASLD model, animals developed marked obesity, reaching ~500 g, compared with ~300 g in RD-fed controls. As expected, in the chronic MASLD model, CDPs treatment did not promote weight gain despite sustained caloric intake, suggesting metabolic effects independent of appetite suppression (Figure 2). Importantly, CDPs significantly reduced VAT accumulation in MASLD-treated animals compared with untreated animals (50 g vs. 70 g), whereas VAT in CTRL groups (10 g) was not affected. VAT expansion is closely linked to insulin resistance and chronic inflammation through the release of pro-inflammatory cytokines and lipotoxic mediators [23]. Therefore, reduced VAT mass suggests improved metabolic health, further supporting the beneficial effects of CDPs treatment in MASLD (Figure 2 and Figure 3).

CDPs improved insulin and glucose sensitivity. Since insulin resistance is a central driver of MASLD progression, promoting dysregulated lipolysis, hepatic gluconeogenesis, and DNL [24], the improved glucose homeostasis observed after CDPs administration supports a beneficial effect on systemic metabolic dysfunction. Notably, these effects became evident during treatment progression, as reduced insulin AUC and improved glucose sensitivity were also observed in healthy animals treated with CDPs, suggesting that their metabolic effects are not restricted to the obese state.

During the 55-week experimental period, the untreated MASLD group had a 25% mortality rate (2/8 rats), whereas all CDP-treated animals survived to study completion (8/8 rats). In addition, one MASLD animal developed a macroscopic myocardial infarction, consistent with systemic complications of chronic obesogenic stress. Consistent with this, serum albumin levels, commonly reduced in advanced hepatic dysfunction and associated with MASLD-related mortality, were restored after CDP treatment. These findings support a direct insulin-sensitizing effect of CDPs. Furthermore, the complete prevention of mortality observed in untreated MASLD animals—associated with myocardial infarction and hypoalbuminemia—suggests a probable cardioprotective effect in the setting of chronic metabolic stress [25]. In our study, MASLD animals exhibited adipocyte hypertrophy, reduced cellular density, low circularity, and increased crown-like structures, consistent with dysfunctional adipose tissue remodeling and chronic inflammation [26]. In contrast, CDP treatment reduced adipocyte size and normalized tissue architecture, suggesting attenuation of pathological adipose expansion. Because hypertrophic adipocytes are strongly associated with lipotoxicity and ectopic lipid deposition [10], these changes are linked to a more favorable metabolic state and contribute to the reduced hepatic steatosis observed in CDP-treated animals. Adipose tissue dysfunction during chronic obesity is also characterized by impaired metabolic plasticity and reduced thermogenic capacity [27]. Therefore, browning of WAT represents a therapeutic potential associated with increased energy expenditure and improved metabolic homeostasis during obesity. This process involves the acquisition of characteristics similar to beige-like adipocytes, including increased mitochondrial content and expression of thermogenic proteins such as UCP1 [9]. Our study also showed that CDP treatment was associated with morphological and molecular features consistent with a beige-like phenotype in WAT.

WAT is characterized by low mitochondrial content and limited basal mitochondrial biogenesis; however, the acquisition of beige-like features is associated with increased mitochondrial activity and oxidative metabolism. In this process, PGC-1α acts as a central regulator of mitochondrial biogenesis by coordinating transcriptional programs involving factors such as NRF1 and TFAM [28]. CDP treatment increased the expression of adipogenic regulators in VAT such as *PPARγ* and *CEBPα*, as well as mitochondrial markers, including *UCP1*, *PGC-1α*, and *NRF1* (Figure 8 and Figure 9). NRF1 regulates genes involved in respiratory chain function and mtDNA replication, both of which are essential for maintaining mitochondrial oxidative phosphorylation capacity [29]. In obesity, mitochondrial biogenesis decreases, leading to reduced expression of NRF1 and PGC-1α. In addition, ATP6v1 expression has been associated with mitochondrial energy synthesis and ATPase activity, whereas Cox4i1, a subunit of complex IV (cytochrome C oxidase), is widely used as a marker of cellular oxidative capacity [28]. Therefore, the increased expression of *PPARγ*, *CEBPα*, *UCP1*, *PGC-1α*, and *NRF1* is consistent with the activation of transcriptional programs associated with mitochondrial function and oxidative metabolism in VAT during chronic MASLD progression.

Because obesity-associated adipocyte hypertrophy increases free fatty acid flux and ectopic lipid accumulation in the liver, the reduction in VAT content with CDP treatment explains the improvement in hepatic steatosis, was accompanied by increased expression of UCP1 and PGC-1α, and by morphological changes consistent with beige-like adipose tissue remodeling [30]. Together, these findings indicate molecular and morphological changes consistent with a beige-like adipose phenotype during the progression of chronic MASLD.

Oxidative stress is a major consequence of MASLD progression, driving lipid peroxidation, inflammation, and hepatocellular injury [31]. CDPs also reversed oxidative stress in the liver. Untreated MASLD animals showed elevated lipid peroxidation and increased expression of antioxidant-response genes (*NRF2*, *GPX1*, and *GCLC*), indicating chronic oxidative stress (Figure 6). In contrast, CDPs reduced lipid peroxidation and normalized glutathione homeostasis, suggesting restoration of redox balance through activation of compensatory antioxidant pathways.

In parallel, CDPs attenuated hepatic steatosis and fibrosis, as evidenced by lower NAS scores, reduced collagen deposition, and decreased hepatic *TGF-β* expression. These effects were associated with suppression of lipogenic genes, including *SREBP-1C* and *ACC1*, and normalization of lipid-transport genes, including *MTTP* and *APOB*. Overall, these findings suggest that CDPs reduce hepatic lipotoxicity and limit inflammatory and profibrotic signaling.

Histopathological analysis of the liver confirmed severe steatosis, inflammatory infiltration, and fibrosis in untreated MASLD animals. MASLD animals treated with CDPs showed less lipid accumulation and fewer inflammatory and fibrotic changes, despite continued exposure to the obesogenic diet. Reduced expression of *TGF-β*, *Fas*, *NFκB*, and *IL-6* further suggests decreased profibrotic signaling (Figure 5).

De novo lipogenesis is a major contributor to hepatic lipid accumulation during MASLD progression [4]; increased expression of *SREBP-1C* and *ACC1* in MASLD animals is consistent with enhanced lipogenic activity (Figure 5). Suppression of these genes after CDPs treatment suggests attenuation of DNL pathways, which may contribute to the reduced hepatic steatosis and lipotoxicity observed in treated animals.

CDP treatment also altered hepatic gene expression involved in fatty acid metabolism and lipid handling [32]. In addition to suppressing the lipogenic genes *SREBP-1C* and *ACC1*, CDP treatment reduced expression of *CD36*, *PSD3*, *MTTP*, *APOB*, and *PPARα*, while partially restoring *ACOX1* expression (Figure 5). Together, these coordinated transcriptional changes are consistent with the marked reduction in hepatic steatosis and suggest an overall restoration of hepatic metabolic homeostasis. In the context of chronic lipid overload, sustained PPARα expression has been proposed as part of an adaptive metabolic response [33,34]. Therefore, its reduced expression following CDP treatment is consistent with attenuation of hepatic metabolic stress accompanying the improvement in liver pathology.

Chronic inflammation is a key driver of MASLD progression, contributing to hepatocyte injury, fibrosis, and steatohepatitis [17,35]. Hepatic *NF-κB* and *IL-6* expression were significantly elevated in untreated MASLD animals and markedly reduced after CDP administration. NF-κB is a central regulator of inflammatory signaling in chronic liver diseases [36], whereas IL-6, contributes to hepatocellular injury, fibrosis, and insulin resistance [37]. In our study, hepatic *NF-κB* and *IL-6* expression were significantly elevated in the MASLD group and reduced following CDPs treatment. These findings suggest that CDPs may attenuate inflammatory signaling during the progression of chronic MASLD, thereby potentially contributing to hepatoprotection.

The findings also suggest that CDPs may influence the transcriptional profile of genes associated with the PI3K/AKT/mTOR pathway. *RASAL2*, a regulator of this pathway and involved in vLDL export, was downregulated in MASLD and linked to increased *MTTP* transcription [18,38,39]. In this study, hepatic *mTOR* and *TSC1/2* expression, as well as *RASAL2* and *MTTP* expression, decreased after CDP treatment, suggesting coordinated transcriptional changes in genes associated with energy homeostasis and lipid metabolism. These observations are consistent with regulatory effects previously observed in vitro in HeLa cells [13]; however, confirmation at the protein level will be necessary to determine whether similar changes occur in the chronic MASLD model.

Beyond their therapeutic efficacy, CDPs exhibited a favorable preclinical safety profile. In this study, no overt signs of toxicity were observed during the 10-week treatment period following intraperitoneal administration, a route selected based on preliminary studies showing greater efficacy than oral delivery. These observations are consistent with previous reports from our group demonstrating a favorable safety profile for the same CDP mixture in both cell and animal models [14,15]. Although encouraging, pharmacokinetic and comprehensive toxicological studies will be required before clinical translation.

In conclusion, CDPs exert beneficial effects on MASLD through multiple complementary mechanisms in both liver and adipose tissue. They improve insulin sensitivity and reduce steatosis, inflammation, oxidative stress, and fibrosis, restoring the expression of MASLD-related dysregulated genes involved in lipid metabolism along with fibrosis and inflammation. In VAT of MASLD animals, crown-like structures and adiposity density were reversed by CDP treatment, increasing expression of genes related to a beige-like adipose tissue phenotype remodeling. These findings suggest that these combined effects improve metabolic homeostasis and survival, supporting the therapeutic potential of CDPs as a strategy for treating MASLD.

## 4. Materials and Methods

### 4.1. Cyclodipeptides Preparation

The *P. aeruginosa* PAO1 strain was grown in Luria–Bertani (LB) broth at 30 °C with shaking. Cell-free supernatant (500 mL) was collected by centrifugation at 10,000× *g* at 20 °C for 10 min. The CDPs were extracted twice with two volumes of ethyl acetate containing acetic acid (0.1 mL/L). The extracts were evaporated to dryness using a rotavapor at 60 °C under vacuum [40]. The residue was dissolved in methanol, and the CDP mixture was enriched using the resin (Chromalite PCG900C; Purolite Life Sciences, Wales, UK), eluting with 40% methanol:water containing TFA (0.001%) and concentrating the solvents by evaporation on a rotavapor at 60 °C under vacuum. Fractions were dissolved in methanol and analyzed by gas chromatography coupled to mass spectrometry (GC-MS). The samples were analyzed by GC-MS (Agilent 7890A, MSD 5975C; Agilent Technologies, Santa Clara, CA, USA), fitted with a capillary column (HP-5MS, 30 m length × 0.25 mm, 0.25 µm film thickness; Agilent Technologies) with helium as the carrier gas. In splitless mode, 1 μL of each sample dissolved in methanol was injected. The mass spectrometer was operated at an ionization voltage of 70 eV and scanned between *m*/*z* 30–500 at 3.9 scans/s. Quantitation was performed using relative peak areas in chromatograms obtained with the MS detector. Compounds were identified by comparison with the mass spectral library (NIST/EPA/NIH, ChemStation, Agilent Technologies Rev. 2023).

### 4.2. Animals

All experimental procedures were conducted in accordance with the GCULA (NIH) and approved by the Institutional Animal Care and Use Committee (IACUC) of the Universidad Michoacana de San Nicolás de Hidalgo (CEIIB-UMSNH/209-2025) and by NOM 062-ZOO-1999 (Ministry of Agriculture, Mexico). Twenty-eight female Wistar rats (6 weeks old) were housed under controlled temperature (20–23 ± 1 °C), humidity (30–70 ± 5%), and 12 h light/dark cycles. The rodents were acclimated for two weeks, and a hypercaloric diet (HFD/CAF) composed of chow pellets (50%), supplemented with vegetable (12.5%) and animal (12.5%) fat sources, and commercial sandwich cookies with cream filling (25%) was used to induce obesity. The regular diet (RD), used as a non-obesogenic diet, was obtained from rodent chow pellets (Rodent Laboratory Chow Purina, Co., Mexico City, Mexico). Animals were randomly assigned to four groups: CTRL (fed a regular diet, RD) *n* = 6, CTRL + CDPs (CTRL rats administered CDPs) *n* = 6, MASLD (rats that developed disease) *n* = 8, and MASLD + CDPs (rats with disease administered CDPs) *n* = 8. Food and water were provided ad libitum. From week 45 onward, CDPs were administered intraperitoneally (5 mg/kg) every three days for 10 weeks.

CDPs (purity > 95%) were dissolved in DMSO:water (1:3) to prepare stock solutions (100 mg/mL). CDPs were administered intraperitoneally to rats at 5 mg/kg, dissolved in pyrogen-free injectable water, every three days for 10 weeks.

### 4.3. Metabolic Tolerance Tests

Metabolic tests were performed before treatment (Week 45) and during CDPs treatment (Week 50 and 55). Rats in all groups underwent an oral glucose tolerance test after a 6 h fast. Glucose was administered orally at 3 g/kg body weight. Blood was drawn from the tail vein at 0, 30, 60, and 120 min to measure plasma glucose concentrations with a glucometer (Accu-Chek Active, Roche DC, Mexico City, Mexico). For the insulin resistance test, animals were fasted for 4 h in the morning. An insulin dose was administered intraperitoneally (0.4 U/kg for CTRL rats and 0.75 U/kg for MASLD rats), and glucose concentrations were monitored. Area Under the Curve (AUC) was estimated. Longitudinal insulin resistance and glucose tolerance test data were analyzed using mixed-effects models (REML), with time and treatment as fixed effects and animal as a repeated factor. Tukey’s test was used for multiple comparisons. Statistical significance was set at *p* < 0.05.

### 4.4. Histopathological Analysis

Liver samples were fixed in 4% paraformaldehyde, dehydrated through graded ethanol and xylene, and sectioned (5–20 μm) for H&E, Oil Red O, and Masson’s trichrome staining. For fluorescence analysis, sections were incubated with propidium iodide and BODIPY, then analyzed on a confocal microscope (Olympus FV1000, Center Valley, PA, USA, 40×). MASLD severity was evaluated using the NAFLD Activity Score (NAS).

Adipose tissue samples were dehydrated in graded ethanol and xylene, sectioned at 8 μm, and stained with H&E. For immunofluorescence analysis, sections were incubated with the UCP1 Alexa Fluor^®^ 546 antibody and MitoTracker Green (Molecular Probes, Invitrogen, Carlsbad, CA, USA) and then visualized on a confocal microscope (Olympus FV1000, 40×). Quantitative image analysis was performed using Fiji/ImageJ 4.2.

### 4.5. RNA Extraction and RT-qPCR

Total RNA was extracted with TRIzol reagent (Invitrogen, Carlsbad, CA, USA) per the manufacturer’s protocol, and quality was assessed with a spectrophotometer (NanoDrop 2000, Thermo Fisher Scientific, Madison, WI, USA). Complementary DNA (cDNA) was synthesized with the iScript Select cDNA Synthesis Kit (Bio-Rad, Hercules, CA, USA) per the manufacturer’s instructions. Quantitative PCR (qPCR) was performed with QuantiNova SYBR Green Supermix (Bio-Rad) on a QuantStudio 3 thermocycler (Applied Biosystems, Thermo Fisher Scientific, Madison, WI, USA) using gene-specific primers (Table S1). Relative gene expression was calculated using the 2^−ΔΔCt^ method and normalized to the *18S* or *ACT* reference genes.

### 4.6. Lipid Peroxidation and Glutathione Analysis

Lipid peroxidation in liver homogenates (500 mg of protein) was determined by the thiobarbituric acid reactive substances (TBARS) assay, and absorbance was measured at 530 nm. Reduced and oxidized glutathione levels were quantified spectrophotometrically using DNTB and glutathione reductase, with absorbance measured at 412 nm.

### 4.7. Statistical Analysis

Statistical analyses were performed using GraphPad Prism version 9.0. Data were presented as means ± SD or SEM. Statistical significance was set at *p* < 0.05. Comparisons among groups were performed using one-way ANOVA with Dunnett’s or Tukey’s post hoc tests, while two-group comparisons were analyzed using an unpaired Student’s *t* test or the Kruskal–Wallis test.

## 5. Patents

This work is a continuation of the research presented in the patent: “Ciclodipéptidos bacterianos con propiedades anti-obesidad y hepato-protectora y sus usos en el tratamiento de la obesidad y enfermedad del hígado graso no alcohólico”. Instituto Mexicano de la Propiedad Industrial. No. De Expediente: MX/a/2024/000709.

## Data Availability

The data supporting the findings of this study are available from the corresponding author upon request.

## Supplementary Materials

The following supporting information can be downloaded at: https://www.mdpi.com/article/10.3390/molecules31142466/s1, Table S1: Oligonucleotides used in this study [41,42,43,44,45,46].

## Abbreviations

The following abbreviations are used in this manuscript:

MASLD	Metabolic dysfunction-associated steatotic liver disease
CDPs	Cyclodipeptides
WAT	White adipose tissue
BAT	Brown adipose tissue
ROS	Reactive oxygen species
HFD/CAF	High-fat/hypercaloric diet
RD	Regular diet
DNL	De novo lipogenesis
H&E	Hematoxylin–eosin staining
VLDL	Very-low-density lipoprotein
TBARS	Thiobarbituric acid reactive substances assay
DTNB	Disulfide 3-carboxy-4-nitrofenilo
*UCP1*	Mitochondrial protein uncoupling protein 1
*TGF-β*	Transforming growth factor beta
*FAS*	Fas cell surface death receptor
*NFκB*	Nuclear factor kappa B subunit 1
*IL-6*	Interleukin-6
*MTTP*	Microsomal triacylglycerol transfer protein
*APOB*	Apolipoprotein B
*RASAL2*	RAS protein activator-like 2
*PPARα*	Peroxisome proliferator-activated receptor alpha
*ACOX1*	Peroxisomal acyl-CoA oxidase gene
*FOXO1*	Forkhead box O1
*SREBP-1C*	Sterol regulatory element-binding protein 1C
*ACC1*	Acetyl-CoA carboxylase 1 gene
*PSD3*	Pleckstrin and Sec7 domain-containing 3
*CD36*	Cluster of differentiation 36
mTOR	Mammalian target of rapamycin
*TSC1*	Tuberous Sclerosis Complex 1 (hamartin)
*TSC2*	Tuberous Sclerosis Complex 2 (tuberin)
AKT	AKT Serine/Threonine Kinase
*NRF2*	Nuclear factor erythroid-derived factor 2
*GPX1*	Glutathione peroxidase 1
*GCLC*	Cysteine-glutamate ligase
*PPARγ*	Peroxisome proliferator-activated receptor γ
*CEBPα*	CCAAT/Enhancer Binding Protein Alpha
*PGC-1α*	Peroxisome Proliferator-Activated Receptor Gamma Coactivator 1-Alpha
*NRF1*	Nuclear Respiratory Factor 1
*CS*	Citrate Synthase
*COX4i1*	Cytochrome c Oxidase Subunit 4 Isoform 1
