# Supplementary material for: Cyclodipeptides Reversed Liver Damage and Adipose Tissue Dysfunction in a Chronic Obesity MASLD Rat Model by Remodeling White Adipocytes Toward a Beige-like Adipocyte Phenotype
Source: Molecules. 2026 Jul 15;31(14):2466. doi: 10.3390/molecules31142466 (PMC13415301; doi:10.3390/molecules31142466)
Supplement: Supplementary file 1 [file molecules-31-02466-s001.zip › molecules-4383031-supplementary.pdf]

Table S1. Oligonucleotides used in this study.

GENE	Tm	Forward	Reverse	REFERENCE
PSD3	54	CATCAGCACATTCCCAGAGT	GTGGGAGTAAAGTGCTTCCT	This work
ACC1	54	TGAGGAGGACCGCATTTATC	AAGCTTCCTTCGTGACCAGA	[1]
PPAR a	55	CATCACCCGAGAGTTCCTAAAG	ATCCAGTTCGAGGGCATTG	[2]
TSC1	56	CCCTCTACCTCCCCAATGGA	GGAGGGTCCGGATCTCATCT	This work
TSC2	57	TGTGACAAGAAACGGCACCT	CACAAGGCCCTCCATGTCTT	This work
FAS	57	TGCTGTGGATCATGGCTGTC	GGTTTCACGAACGCTCCTCT	This work
GPX1	57	CCCGGGACTACACCGAAATG	CGGGTCGGACATACTTGAGG	This work
GCLC	57	TTACTGAATGGCGGCGATGT	CGGCGTTTCCTCATGTTGTC	This work
NRF2	57	TCTGACTCCGGCATTTCACT	GGCACTGTCTAGCTCTTCCA	This work

ACOX1	57	TGTCTGTCACTTCTGTCGCC	CGGACTGCCATCCAAGATGT	[3]
RASAL2	57	GGGCAGAGAGCTGTCAGTTT	AGAGGTCCCAATTTTGCCACA	This work
TGF B1	57	AGTGGCTGAACCAAGGAGAC	CAGGAAGGGTCGGTTCATGT	This work
CD36	58	GGCTAGCTGATTACTTCTGTGTAGT	CCTCCTCGTGCAGCAGAATCA	This work
APOB	58	CTGCTGAATGGTGCGCAAAC	CAGAGGGCTTTGCCACTAGC	This work
ACT	60	CATTGCTGACAGGATGCAGAAGG	TGCTGGAAGGTGGACAGTGAGG	[4]
SREBP-1C	59	CGC TAC CGT TCC TCT ATC AAT GAC	AGT TTC TGG TTG CTG TGC TGT AAG	[5]
MTTP	60	AAGGCCAATATGGACATCCAGGGT	TGGTTATTACCACAGCCACCCGAT	[6]
UCP1	56	ACACTGTGGAAAGGGACGAC	TACTGTCAGCTCTTGTTGCC	This work
PGC1a	60	GTACAACAATGAGCCCGCGAACATAT	CAGTGGTCACGTCTCCATCTGTC	This work
CEBPA	60	TTCGGGTCGCTGGATCTCTA	TCAAGGAGAAACCACCACGG	This work

COX4i1	60	TCTACTTCGGTGTGCCTTCG	CCACATCAGGCAAGGGGTAG	This work
CS	60	ATTGGGGCAATTGACTCTAAGC	CCCTGCCAGCCCATTCATAG	This work
NRF1	60	CATGGACCATCAGCAAAGCC	ACAACGTAAGCTCTGCCTGG	This work
ATP6v1g3	60	TCTATTGGCTCAGAGGACGC	TGCCTTTTGGACTGACAGTGG	This work
PPARγ	60	AGGGACTCGAGGAGGTCAAGAAG	ATTCCGAAGTTGGTGGGCCAG	This work
NFkB	60	TCCCACAAGGGGACATTAAGC	TCCACCAGCTCTTTGATGGTC	This work
mTOR	60	TTCACGATCGGAGCCAACAA	GCAAATCGTGGTGGCTCTTC	This work
AKT	60	TCAGGTGCTGAGGAGATGGA	CTTGGCAACGATGACCTCCT	This work
Il-6	60	CTTGGGACTGATGCTGGTGACA	GCCTCCGACTTGTGAAGTGGTA	This work
FOXO1	60	CAATTCGCCACAATCTGTCCC	TCTTGCCTCCCTCTGGATTG	This work
18S	60	GCAAATTACCCACTCCCGAC	CCGCTCCCAAGA TCCAACTA	This work

References:

1. Li, M., et al., *Effect of Octreotide on Hepatic Steatosis in Diet-Induced Obesity in Rats.* PLoS One, 2016. **11**(3): p. e0152085.
2. Elekofehinti, O.O., & Akinjiyan, M. O., *Effects of momordica charantia silver nanoparticles on the expression of genes associated with lipid metabolism and nephrotoxicity in stretozotocin-induced rats.* Nigeria Journal of Biotechnology, 2020. **37**: p. 126-133.
3. Ebihara, C., et al., *Different sites of actions make different responses to thiazolidinediones between mouse and rat models of fatty liver.* Scientific Reports, 2022. **12**(1): p. 449.
4. Tian, Y., et al., *Mesenchymal stem cells improve mouse non-heart-beating liver graft survival by inhibiting Kupffer cell apoptosis via TLR4-ERK1/2-Fas/FasL-caspase3 pathway regulation.* Stem Cell Res Ther, 2016. **7**(1): p. 157.
5. Kaviarasan, K. and K.V. Pugalendi, *Influence of flavonoid-rich fraction from Spermacoce hispida seed on PPAR-alpha gene expression, antioxidant redox status, protein metabolism and marker enzymes in high-fat-diet fed STZ diabetic rats.* Journal of basic and clinical physiology and pharmacology, 2009. **20**(2): p. 141-158.
6. Dettlaff-Pokora, A., T. Sledzinski, and J. Swierczynski, *Up-Regulation Mttp and Apob Gene Expression in Rat Liver is Related to Post-Lipectomy Hypertriglyceridemia.* Cell Physiol Biochem, 2015. **36**(5): p. 1767-77.
